# Label-free isolation of heterogeneous breast cancer cell populations *via* insulator-based dielectrophoresis

**DOI:** 10.1039/d6an00976j

**Published:** 2026-11-09

**Authors:** Gürhan Özkayar, Ismene N. Usman, Ece Yakın, Jaco Kraan, Kristen David, Duco Bosma, John W. M. Martens, Peter ten Dijke, Georg R. Pesch, Pouyan E. Boukany

**Affiliations:** a Department of Chemical Engineering, Delft University of Technology Delft the Netherlands p.e.boukany@tudelft.nl; b Department of Medical Oncology, Erasmus MC Cancer Institute, Erasmus University Medical Center Rotterdam the Netherlands; c Oncode Institute and Department of Cell Biology, Leiden University Medical Center Leiden the Netherlands; d Institute of Chemical, Environmental and Bioscience Engineering, TU Wien Vienna Austria

## Abstract

Circulating tumor cells (CTCs) are valuable biomarkers for cancer diagnosis and monitoring, yet their isolation from blood remains challenging due to their phenotypic heterogeneity and rarity. Label-free microfluidic technologies offer a promising alternative to affinity-based approaches by exploiting intrinsic biophysical differences between cell types. Here, we developed a microfluidic platform for label-free cell separation based on insulator-based dielectrophoresis (iDEP). The microfluidic device employs an array of triangular insulating structures that generate strong electric field gradients in response to an externally applied alternating current (AC) electric field, enabling selective isolation of breast cancer cells from blood cells based on their dielectric properties. Hydrodynamic focusing is used to confine the sample stream and precisely control cell trajectories within the separation region. Numerical simulations were performed to optimize the electric field distribution and fluid flow characteristics within the device. Experimental validation using breast cancer cell lines (mesenchymal-like MDA-MB-231 cells and epithelial-like MCF-7 cells) spiked into peripheral blood mononuclear cells (PBMCs) demonstrated selective dielectrophoretic deflection of cancer cells while PBMCs largely followed the central streamline. Approximately 98% of the cancer-cell flux detected across all outlets exited through the CTC outlets with a purity above 65% in 1 : 1 spike-in experiments. The ability to enrich both mesenchymal-like and epithelial-like breast cancer cells demonstrates the potential of the platform to address phenotypic heterogeneity which is a key challenge in label-free isolation of CTCs. The proposed system provides a simple label-free approach to separate heterogeneous cell populations and represents a promising tool for microfluidic liquid biopsy enrichment applications.

## Abstract 2

A hydrodynamically focused insulator-based DEP microfluidic platform enables label-free enrichment of heterogeneous breast cancer cells from PBMCs. Ratchet-like insulators create non-uniform electric fields that direct cancer cells to CTC outlets.

## Graphical abstract

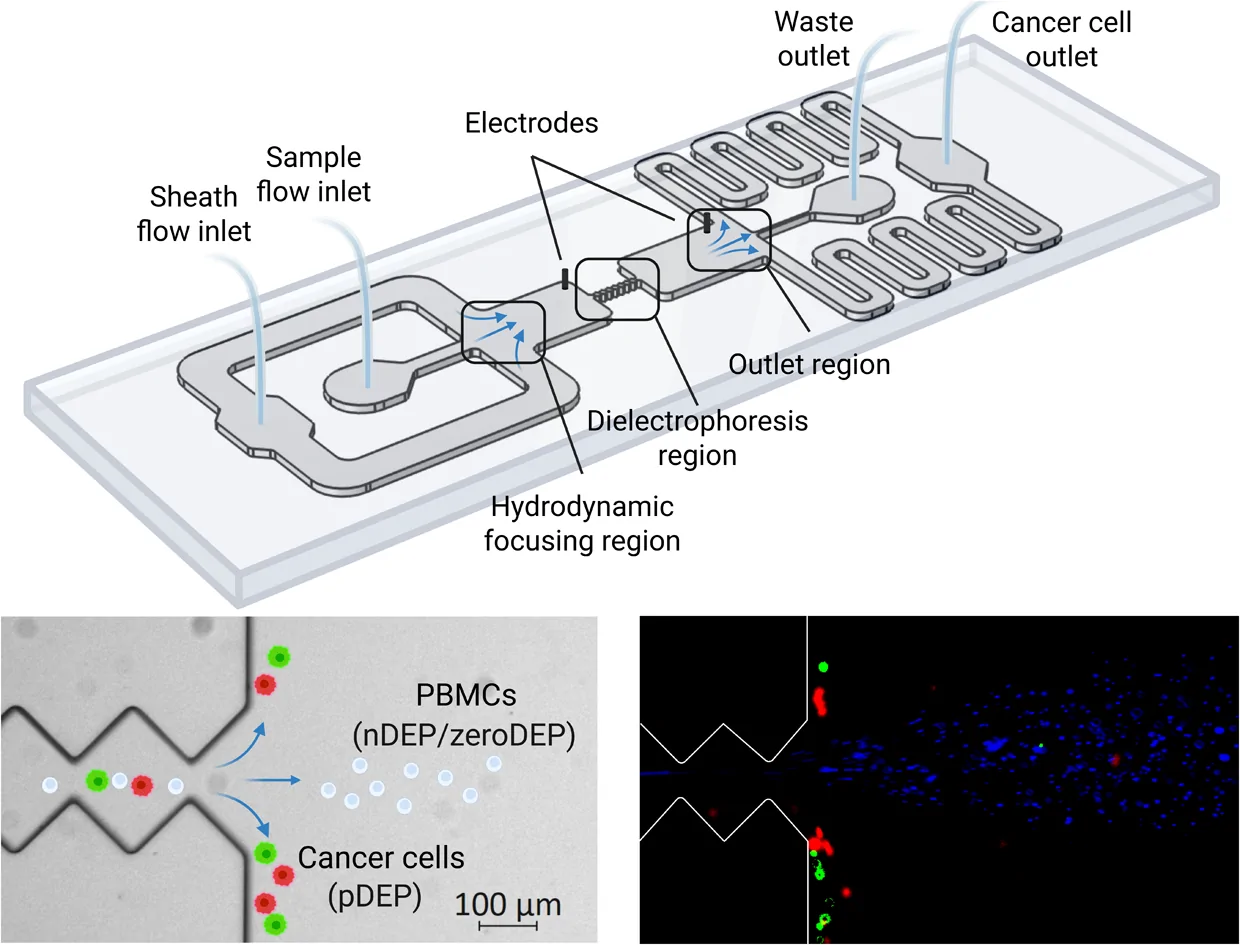

## Introduction

Liquid biopsy has emerged as a minimally invasive approach for detection and characterization of cancer-associated biomarkers, including circulating tumor cells (CTCs).^1,2^ CTCs provide unique insight into tumor heterogeneity, disease progression, and response to treatment.^3,4^ However, the biological and biophysical heterogeneity of CTCs poses a major challenge to their reliable isolation and downstream analysis.^5,6^ This challenge is particularly relevant in breast cancer,^7^ which is characterized by substantial heterogeneity and is considered one of the most heterogeneous malignancies affecting women worldwide.^8^ Variations in cellular phenotype, morphology, deformability, and dielectric properties, together with dynamic epithelial–mesenchymal transition (EMT) states, have been frequently associated with metastatic progression, therapeutic resistance, and patient-specific treatment response.^9,10^ Accordingly, the importance of addressing CTC heterogeneity has been increasingly emphasized in numerous studies in cancer biology^11–13^ and microfluidics-based diagnostics.^14–16^ Developing isolation technologies capable of enriching heterogeneous CTC populations while preserving their diverse cellular characteristics is therefore a key requirement for investigating tumor heterogeneity and enabling subsequent analysis.^17^

Microfluidic platforms have been extensively explored for the isolation and characterization of CTCs,^18^ owing to their potential for scalable processing, compatibility with clinical workflows, and ability to handle clinically relevant sample volumes. Microfluidic strategies for CTC isolation can broadly be classified into affinity based and label-free approaches.^19^ Affinity-based methods rely on antibody recognition of surface biomarkers, such as epithelial cell adhesion molecule (EpCAM), leukocyte common antigen (CD45), enabling specific and non-specific enrichment of epithelial-like tumor cells.^20^ Representative platforms include the clinically validated CellSearch System,^21^ as well as the AdnaTest^22^ and PowerMag System,^23^ which utilize immunomagnetic separation principles for CTC enrichment. Despite their clinical utility, these approaches remain inherently biased toward specific phenotypic subpopulations, captured CTCs lose their viability after fixation,^24^ and may fail to capture the full CTC spectrum, such as clinically relevant CTC subpopulations that downregulate epithelial markers during epithelial–mesenchymal transition (EMT).^25–27^

Label-free microfluidic platforms on the other hand do not rely on molecular markers for heterogeneous CTCs^28^ and CTC clusters.^29^ They exploit intrinsic biophysical and electrical differences between cell populations, including size, deformability, density, and dielectric properties.^30^ Among them, dielectrophoresis (DEP) is particularly attractive due to its sensitivity to subtle variations in cellular electrical properties^31^ and its capacity for continuous, tunable, and marker-independent cell separation.^32–34^ Importantly, DEP can discriminate between cell populations with overlapping size distributions by exploiting differences in their frequency-dependent dielectric properties, providing an additional separation dimension beyond size-based approaches alone.^35^

DEP has been effectively implemented using electrode-based (eDEP)^36–38^ or insulator-based (iDEP)^39–41^ architectures; while eDEP relies on integrated microelectrodes to generate non-uniform electric fields, iDEP uses insulating microstructures to shape the electric field generated by external electrodes, avoiding electrodes within the separation region. Compared with eDEP, iDEP avoids electrodes within the separation region, thus reducing electrode fouling, electrochemical reactions, and bubble formation.^42^ It also enables relatively simple device fabrication using established photolithography and soft-lithography techniques.^43^

Previous DEP studies have predominantly evaluated individual cancer cell lines or relatively homogeneous target populations under controlled experimental conditions, such as prostate cancer cells,^44^ melanoma cells,^45,46^ and breast cancer cells including MCF-7 cells^47^ and MDA-MB-231 cells.^48–50^ Furthermore, relatively few studies have combined hydrodynamic focusing with DEP to improve manipulation consistency of biological samples such as platelets,^51^ serum,^52^ and cancer cell lines^53^ under continuous-flow conditions. In particular, the manipulation of phenotypically heterogeneous cancer-cell populations using strengths of both hydrodynamic focusing and iDEP remains comparatively underexplored. This gap highlights the need to develop and evaluate label-free DEP platforms capable of manipulating heterogeneous cancer-cell populations.

In this study, a continuous-flow iDEP microfluidic platform was developed to investigate whether hydrodynamic focusing combined with iDEP could enable label-free manipulation and enrichment of phenotypically contrasting epithelial-like and mesenchymal-like breast cancer-cell populations from a PBMC background under fixed electrical and hydrodynamic conditions. To provide controlled initial conditions for DEP-mediated migration, hydrodynamic focusing was integrated to confine cells within a narrow lateral distribution before entering the DEP region. To model phenotypic heterogeneity, we used two widely used breast cancer cell models representing mesenchymal-like (MDA-MB-231)^54,55^ and epithelial-like (MCF-7)^56,57^ characteristics. The device incorporates ratchet-like insulating structures designed to generate strong localized electric field gradients, enabling modulation of cancer cell trajectories while minimally affecting blood cell populations. Combined numerical analysis and experimental validation show that the platform achieves high-cancer cell manipulation rates within heterogeneous suspensions.

## Materials and methods

The experimental methodology used to develop and evaluate the continuous-flow iDEP platform is described below, including device fabrication, numerical modelling, cell preparation, experimental procedures, and quantitative performance analysis.

### Device design and operating principle incorporating DEP theory

The microfluidic device was designed to enable continuous, label-free separation of cancer cells from peripheral blood mononuclear cells (PBMCs) using insulator-based dielectrophoresis (iDEP). As shown in Fig. 1a and b, the device consists of two inlets (a sample inlet and a sheath flow inlet) and two outlets (a waste outlet and a CTC outlet). A mixture of cells is introduced through the sample inlet. The main microchannel comprises three functional regions: a hydrodynamic focusing section to confine the sample stream, a constriction region incorporating triangular ratchet-like insulating structures to generate non-uniform electric field, and an outlet section for separated stream collection. A sheath flow from side inlet channels is applied at equal rates to narrow the sample stream towards the center of the channel. Hydrodynamic focusing is crucial for maintaining a narrow sample stream inside the constriction channel, ensuring uniform exposure to DEP and keeping PBMCs on the center streamline which routes to the waste outlet (Fig. 1c).

**Fig. 1:**
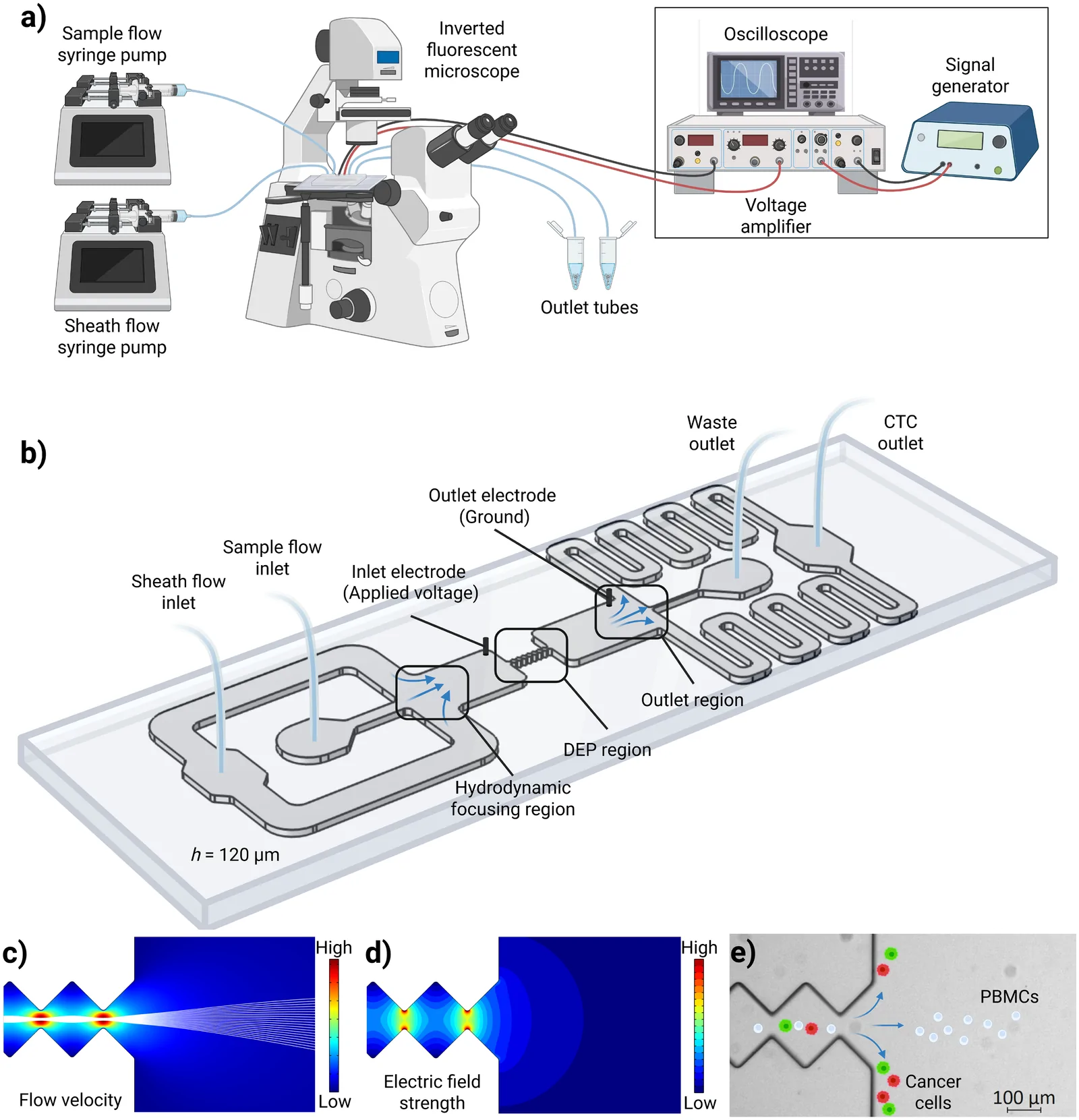
Schematic representation of the microfluidic device and experimental setup, together with the operating principle of iDEP. (a) Fluidic connections are established polytetrafluoroethylene (PTFE) tubing (0.5 mm i.d.; blue), and flow is controlled using two syringe pumps dedicated to sample and sheath streams. The microfluidic chip is mounted on an inverted fluorescence microscope for image acquisition. The electrical setup comprises a signal generator, a voltage amplifier, and an oscilloscope for signal monitoring. (b) The microfluidic device has a channel height of 120 μm, a width of 2.25 mm in the main channel, and a constriction width of 50 μm at DEP region. An electric field is generated across the obstacle array by applying a potential difference using two platinum electrodes (black cylinders) spaced 5.5 mm apart. The outlet electrode was held at ground (*V* = 0), while the inlet electrode was driven by the applied AC voltage. (c) Simulated fluid velocity distribution in the constriction channel exit. White streamlines indicate the focused sample stream. The maximum fluid velocity occurs between the tips of the triangular obstacles in the constriction region. (d) Simulated electric field distribution illustrating the high-field regions formed at the tips of the insulating triangular structures due to the non-uniformity of the applied electric field. (e) Schematic illustration of particle manipulation in the actual PDMS channel. The dielectrophoretic force acts perpendicular to the flow direction; cancer cells (green and red) experiencing pDEP are attracted toward the high-field regions at the obstacle tips and are deflected toward trajectories closer to the sidewalls. At the selected operating frequency, PBMCs (white) experience limited DEP forces and remain along the central streamline. In the absence of DEP forces, all cells follow the hydrodynamically focused center path and exit through the waste outlet (not shown here). Cell trajectories are determined by the balance between hydrodynamic drag and dielectrophoretic forces, which can be tuned through the applied voltage and flow rates.

As the focused flow enters the constriction channel, the constrictions generate strong electric field gradients when an AC voltage is applied across the channel (Fig. 1d). Platinum electrodes (0.38 mm diameter; Goodfellow, PT00-WR-000143) are inserted through designated through-holes on both sides of the constriction region, spaced 5.5 mm apart, allowing voltage application without embedding metal micro-electrodes within the actual DEP manipulation region. The electrode locations were selected to maintain clearance from the hydrodynamically focused sample stream and the outlet junction, minimizing perturbation of the intended flow and cell trajectories. The majority of the voltage drops across the sawtooth geometry, therefore the exact placement of the electrodes within the inlet and outlet chamber does not significantly change the electric field distribution. The non-uniform electric field generates DEP forces that redirect positively DEP-experienced cancer cells towards the tips. As a result, they are moved onto streamlines directed toward the cancer cell outlet. In contrast, PBMCs experience weaker, and negligible DEP forces, remain in their original streamlines and proceed along the central axis, directed towards the waste outlet (Fig. 1e). This allows sorting into distinct outlets based on cells’ DEP response.

The time-averaged DEP force *F*_DEP_, acting on a spherical particle of radius *r*, suspended in a medium with a relative permittivity *ε*_m_, is given by:

1 *F*_DEP_ = 2π*r*^3^*ε*_m_*ε*_0_Re[*K*(*ω*)]*∇*|*E*|^2^

where *ε*_0_ is permittivity of vacuum, *E* is the amplitude (rms) of the electric field, *∇* is the gradient operator, *ω* = 2π*f* is the angular frequency of the applied signal, *f* is the frequency of the applied field, and Re[*K*(*ω*)] is the real part of the Clausius–Mossotti factor. This factor is defined as2
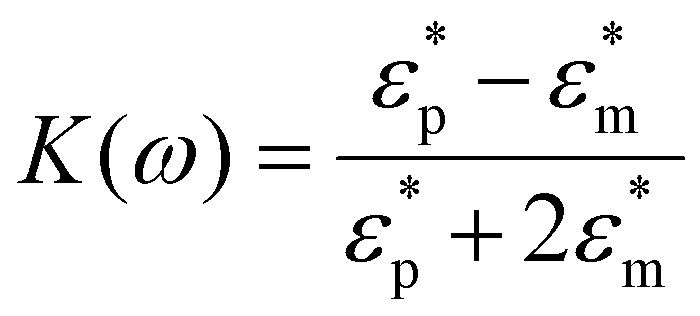
with the complex permittivities 
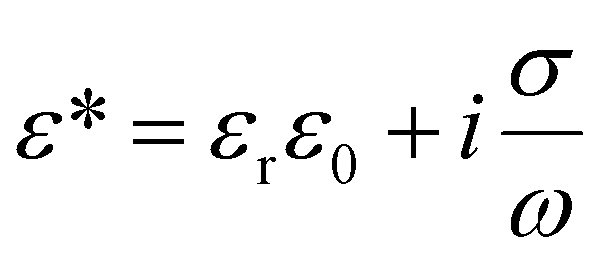
 of the particle (subscript p) and the surrounding medium (subscript m). Here, *ε*_r_ and *σ* are the relative permittivity and conductivity of the particle and medium, *ε*_0_ is the permittivity of free space, and 
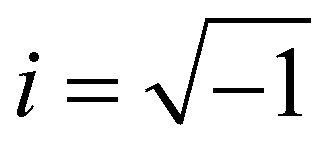
 is the imaginary number. The sign and magnitude of Re[*K*(*ω*)] determine the direction and strength of the DEP force. When Re[*K*(*ω*)] > 0, positive DEP (pDEP) occurs, cells are attracted to regions of high electric field intensity. When Re[*K*(*ω*)] < 0, negative DEP (nDEP) occurs, cells are repelled from high-field regions and migrate to low electric field regions. At Re[*K*(*ω*)] = 0, zero DEP occurs, the particle does not experience a net DEP force, although it is still subject to fluidic drag.

Due to the frequency dependence of the complex permittivity, the Clausius–Mossotti factor and therefore the DEP behavior of a cell (pDEP or nDEP) depend on the frequency of the applied field. While homogeneous particles usually experience only a single change in the DEP behavior with frequency (one *dispersion*), mammalian cells are usually modeled using the single-shell model^32^ and experience two dispersions, one in the kHz range and one in the MHz range.^58,59^ The frequency of the first dispersion is slightly lower for most cancer cells than for white blood cells, as it is inversely related to the cell membrane capacitance.^60^ Cancer cells typically exhibit higher membrane capacitance due to their more complex and highly folded (wrinkled) membrane morphology, resulting in a lower first dispersion frequency.^60,61^

Fig. 2 shows the value of Re[*K*(*ω*)] for white blood cells and two breast cancer cell lines (MCF-7 and MDA-MB-231) at a medium conductivity of 2 mS m^−1^. Low-conductivity media are commonly employed in DEP systems to reduce Joule heating associated with the application of high electric fields.^62^ 8 kHz was selected as the operating frequency for selective pDEP-based isolation of cancer cells from PBMC populations. This frequency is consistent with the conductivity dependent crossover behavior of white blood cells reported in a previous study,^63^ where a crossover frequency of 271 kHz was observed at a medium conductivity of 70 mS m^−1^, corresponding to approximately 8 kHz under the lower conductivity conditions (2 mS m^−1^) used in the present study. Under these conditions, cancer cells exhibit pDEP behavior, whereas most white blood cells display only weak pDEP (with Re[*K*(*ω*)] values three-fold lower than those of the cancer cells) or transition toward nDEP.

**Fig. 2:**
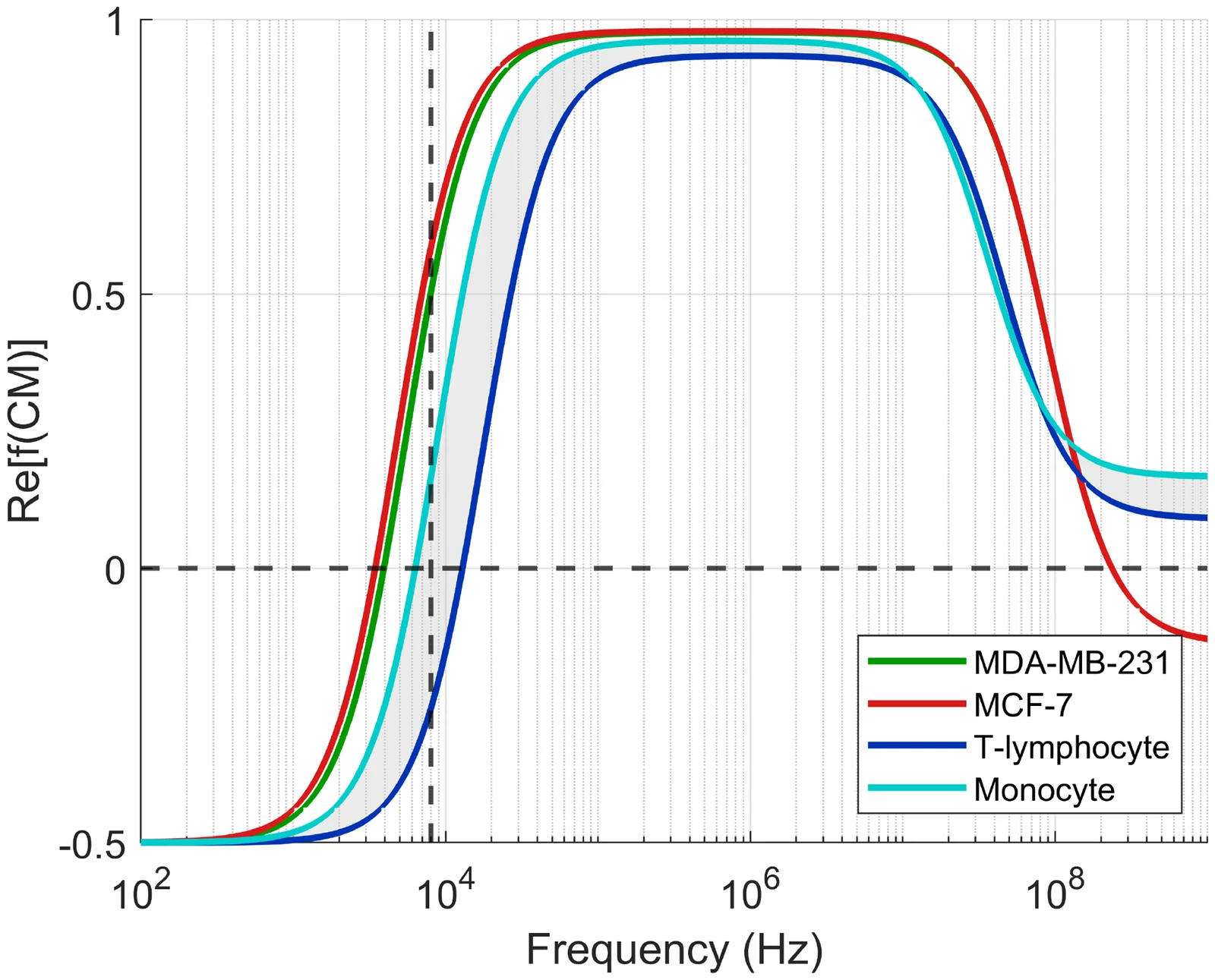
Real part of the Clausius–Mossotti spectra of the MDA-MB-231, MCF-7 breast cancer cell lines,^64^ and major PBMC subpopulations^65^ calculated at a medium conductivity of 2 mS m^−1^ using MyDEP.^66^ The grey shaded region indicates the DEP response envelope between T-lymphocytes and monocytes, the dominant PBMC populations. The dashed vertical line marks the selected operating frequency (8 kHz), while the dashed horizontal line corresponds to Re[*K*(*ω*)] = 0. At 8 kHz, cancer cells exhibit pDEP having Re[*K*(*ω*)] > 0.5, whereas PBMC subpopulations show weak pDEP (Re[*K*(*ω*)] ≈ 0.16) or nDEP (Re[*K*(*ω*)] ≈ −0.25).

At the end of the main channel, the outlets are divided into a central waste outlet, which collects the unmanipulated PBMCs, and two side outlets that merge to collect the deflected cancer cells. The effectiveness of this mechanism relies on a careful balance between fluidic drag and DEP force. Hydrodynamic focusing plays a crucial role in confining the sample flow, ensuring that PBMCs remain within the central streamline and reach the waste outlet, while manipulated cancer cells are displaced toward the sidewalls and sorted accordingly.

To verify that the proposed device geometry and operating conditions are sufficient for effective dielectrophoretic manipulation, two-dimensional and three-dimensional finite-element simulations were performed. Simulations were used to evaluate the flow velocity distribution, electric field strength, electric field gradient, and the uniformity of these fields throughout the channel height. Details of the computational model, boundary conditions, mesh, and representative simulation results are provided in the SI S1 (Fig. S1 and S2).

Hydrodynamic flow focusing behaviour was characterized to establish the baseline cell trajectories without DEP. The numerical flow fields (Fig. S3a and b) suggest a 2.5× confinement of cell-carrying streamlines by the sheath flow, resulting in a narrow focused stream entering the DEP region. Experimental streamines (Fig. S3c and d) agree well with the simulated ones.

### Microfluidic device fabrication and experimental setup

The microfluidic devices were fabricated using standard soft lithography techniques.^67^ The master mold was fabricated on a 4 inch silicon wafer in a cleanroom environment using a μMLA Laser Writer (Heidelberg Instruments). The resulting microfluidic channel height was 120 ± 5 μm. Polydimethylsiloxane (PDMS, Sylgard 184, Dow Corning) was mixed in a 10 : 1 ratio (base to curing agent), degassed under vacuum, and poured over the mold. After curing at 70 °C for 2 hours, the PDMS layer was peeled off and access holes for inlets, outlets, and electrode connections were punched using biopsy punches. The PDMS device was irreversibly bonded to a glass slide following oxygen plasma treatment using a plasma cleaner (PDC-002-CE, Harrick Plasma, USA) to seal the microchannels. The completed chip was placed in an oven at 70 °C for at least 30 minutes to enhance bonding strength before use.

The experimental setup (Fig. 1a) consisted of two identical syringe pumps (Pump 11 Elite, Harvard Apparatus, USA) that independently drove the sample and sheath flows into their designated inlets at controlled flow rates. A sheath-to-sample flow-rate ratio of 2 : 1 (10 μL min^−1^ and 5 μL min^−1^, respectively) was selected based on preliminary experimental testing to achieve stable flow and consistent hydrodynamic focusing before the DEP region. Before sample introduction, fluidic system was primed and visually inspected to confirm continuous, bubble-free flow. During operation, hydrodynamic focusing and cell trajectories were visually monitored.

An AC signal was applied across the device electrodes using a signal generator (Rigol DG4062, Rigol Technologies, PRC), with the operating frequency and voltage selected based on the simulations described in the SI. The signal was subsequently amplified 200-fold using a high-voltage amplifier (Trek PZD700A, Advanced Energy Industries, USA). Electrical stability was monitored continuously using an oscilloscope (Rigol DS7014, Rigol Technologies, PRC) to verify waveform integrity, applied frequency, and voltage amplitude.

### Microscopy setup, video recording and post processing

Real-time monitoring of cell motion was performed using an inverted fluorescence microscope (Axio Observer Z1/7, Zeiss GmbH, Germany) equipped with a Colibri 7 illumination system. For fluorescence imaging of GFP, mCherry, and Calcein Blue, LED modules with peak wavelengths of 475 nm, 567 nm, and 385 nm were used in combination with 38 HE GFP (ex: 488 nm, emm: 509 nm), 56 HE GFP/DsRed (ex: 587 nm, emm: 610 nm), and 49 DAPI (ex: 400 nm, emm: 452 nm) filter sets. Time-lapse image sequences were acquired using a 10× air objective (Plan-Apochromat 10×/0.45 M27) and an ORCA Flash 4.0 V2 sCMOS camera (Hamamatsu Photonics, Japan) at a resolution of 2048 × 2048 pixels.

Before each experiment, the same imaging configuration was maintained for all outlet measurements. Although detection efficiency across the full 120 μm channel height was not independently calibrated, the ImageJ analysis was optimized to detect both sharply focused and visibly defocused fluorescent cells using contrast enhancement and filtering. Image acquisition was initiated only after the hydrodynamic focusing pattern and outlet flow distribution had visually stabilized. Following application of the electric field, recordings were obtained after the DEP-induced cell trajectories showed no obvious transient changes. Steady operation was assessed from the stability of the focused stream and cell trajectories during the observation period.

For each experimental condition, 50 fluorescence frames were recorded with an exposure time of 5 ms using Zen 3.13 software and stored in .czi format. For two-channel experiments, the average interval between successive frames of the same channel was approximately 1.4 s, whereas for three-channel PBMC spike-in experiments it was approximately 2.6 s because the green, red, and blue channels were acquired sequentially. The acquired images were subsequently processed in ImageJ (v1.54p, National Institutes of Health, USA). A detailed image processing workflow is provided in the SI S2. The results of the post processing and cell counting are presented in the Results and discussion section.

### Cell culture, PBMC isolation, and sample preparation

Two malignant human breast cancer cell lines were used in the experiments: MDA-MB-231 cells expressing LifeAct-GFP and MCF-7 cells constitutively expressing cytoplasmic mCherry. As these cell lines were genetically modified to express fluorescent markers, no additional staining was required. They were cultured in Dulbecco's modified Eagle's medium (DMEM, Gibco) supplemented with 5% fetal bovine serum (FBS, Gibco). Cells were maintained at 37 °C in a humidified incubator with 5% CO_2_ and subcultured at least twice per week. All experiments were conducted using cells with passage numbers not exceeding 20 (P20) to minimize passage-related changes in cellular characteristics. Prior to each experiment, the average cell diameter of both cell lines was measured to be approximately 14 μm using an automated cell counter (TC20, Bio-Rad Laboratories, Inc., USA).

PBMCs were isolated from whole blood collected from healthy human donors at Erasmus MC, Rotterdam, following ethical approval and informed consent. Whole blood was diluted 1 : 1 with sterile phosphate-buffered saline (PBS) and gently layered over Ficoll-Paque Plus (GE Healthcare) in 50 mL conical tubes. Samples were centrifuged at 400*g* for 30 minutes at room temperature with the brake off. After centrifugation, the buffy coat containing the PBMC layer was carefully collected, washed twice with PBS, and centrifuged at 300*g* for 10 minutes to remove residual Ficoll and plasma components. The PBMC were then stained with either Calcein Green or Calcein Blue (Thermo Fisher Scientific, Waltham, MA) at a final concentration of 2 μM to enable fluorescent labeling depending on the experiment type. Following staining, the cells were washed with PBS, and the resulting cell pellet was resuspended in DEP buffer for subsequent microfluidic experiments. Cell concentration and viability were assessed using trypan blue exclusion and an automated cell counter (TC20, Bio-Rad Laboratories, Inc., USA).

To ensure appropriate dielectric contrast and maintain cell viability, a low-conductivity DEP buffer was prepared by dissolving 8.5% (w/v) sucrose (≥99.5%, Sigma-Aldrich, USA) and 0.3% (w/v) dextrose (≥99.5%, Sigma-Aldrich, USA) in deionized (DI) water (obtained from Milli-Q IQ 7000, Ultrapure Water Purification System, Merck, Germany). A small volume of 1× phosphate-buffered saline (PBS) was added to adjust the conductivity to the target value of 2 mS m^−1^, which is suitable for effective DEP operation. The buffer was sterile-filtered using a 0.22 μm filter and was used fresh for each experiment.

Prior to each experiment, cancer cells and PBMCs were washed twice with DEP buffer and resuspended at the desired concentrations. For single-cell-type experiments, MDA-MB-231 and MCF-7 cells were prepared separately at a concentration of 1 × 10^6^ cells per mL in DEP buffer. For co-culture experiments, MDA-MB-231 and MCF-7 cells were mixed at a 1 : 1 ratio while maintaining a total cancer cell concentration of 1 × 10^6^ cells per mL. To evaluate separation performance under biologically relevant conditions, spike-in PBMC samples were prepared by mixing cancer cells and PBMCs at a 1 : 1 ratio, corresponding to final concentrations of 1 × 10^6^ cancer cells per mL and 1 × 10^6^ PBMCs per mL. For all experimental conditions, 1 mL of sample suspension was prepared and introduced into the microfluidic device.

## Results and discussion

The performance of the continuous-flow iDEP platform is evaluated below, progressing from individual breast cancer cell populations to heterogeneous cancer-cell mixtures and, subsequently, cancer-cell enrichment in a PBMC background.

### Trajectory modulation of heterogeneous cancer cells under iDEP actuation

To evaluate the separation capability of the iDEP platform, the cell trajectory behavior was investigated under conditions with and without an applied electric field in the DEP region. Hydrodynamic focusing confined MDA-MB-231 cells, MCF-7 cells, and PBMCs to the central streamline of the main microchannel prior to entering the DEP region consisting of a constriction channel incorporating triangular insulating sidewall structures. In the absence of an electric field, all cell populations predominantly followed trajectories toward the waste outlet after exiting the DEP region. This behavior indicates that hydrodynamic drag forces govern cell motion when electric field gradients are absent (Fig. 3a–d).

**Fig. 3:**
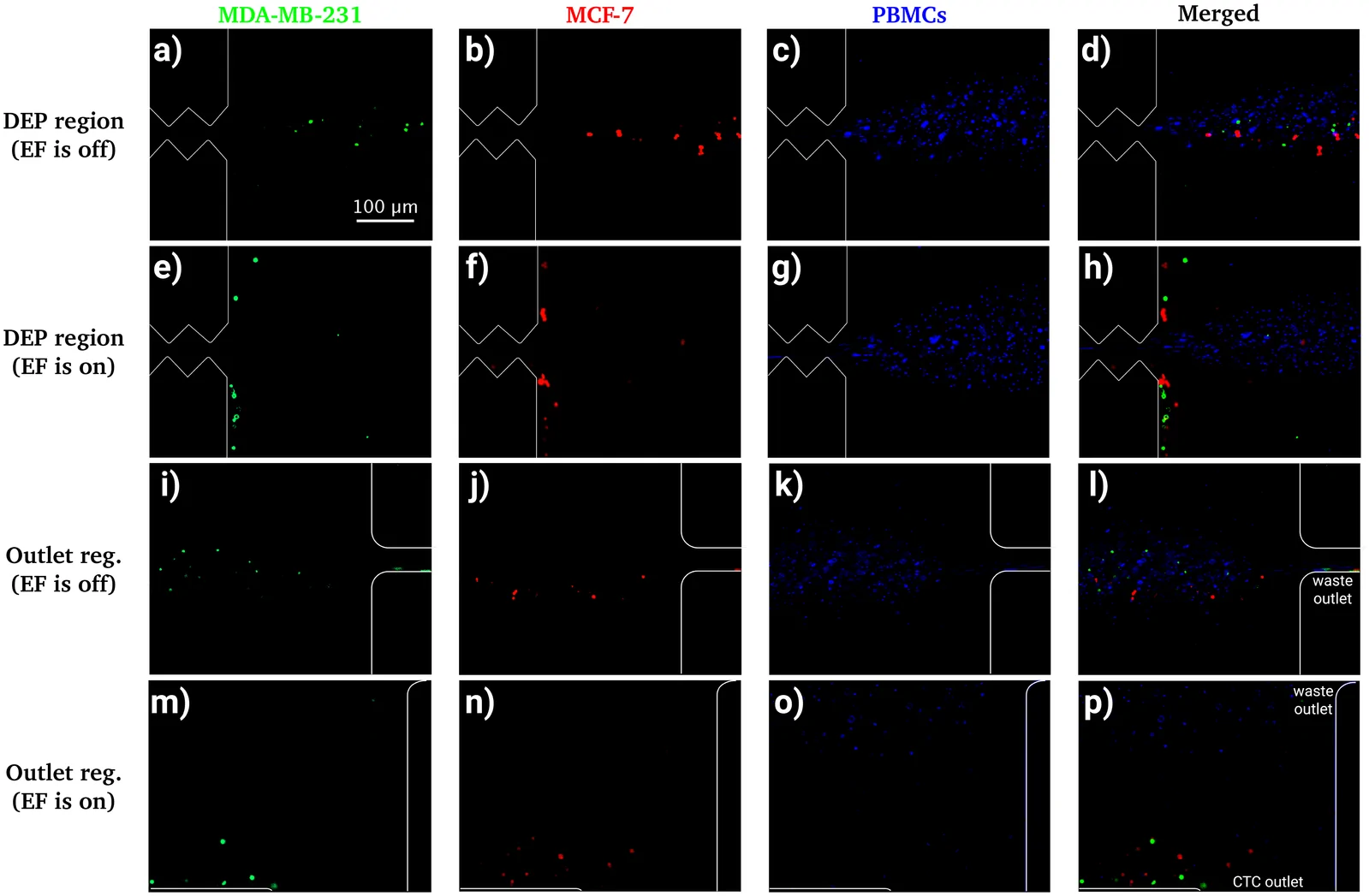
Trajectory modulation of heterogeneous cancer cells and PBMCs under hydrodynamic focusing and dielectrophoretic (DEP) actuation for solo experiments. Following hydrodynamic focusing, cells are confined to the central streamline upon exiting the constriction region and predominantly follow trajectories toward the downstream waste outlet in the absence of an electric field (EF off). Fluorescence microscopy images showing the displacement behavior of (a) MDA-MB-231-LifeAct GFP breast cancer cells, (b) MCF-7-mCherry breast cancer cells, (c) PBMCs stained with Calcein Green, and (d) merged fluorescence images under EF-off conditions. Under these conditions, all cell populations remain broadly distributed around the central streamline and are mainly directed toward the waste outlet. Upon application of an AC electric field (210 V_RMS_, 8 kHz), cancer cells experience positive dielectrophoresis (pDEP) near the insulating obstacle tips, resulting in lateral migration. Fluorescence images of (e) MDA-MB-231 and (f) MCF-7 cells demonstrate DEP-induced displacement toward streamlines closer to the channel sidewalls, ultimately redirecting the cells into the designated CTC outlet streams. (g) PBMC trajectory behavior under identical DEP conditions shows only weak lateral displacement near the constriction exit, indicating a limited dielectrophoretic response. (h) Merged fluorescence images under DEP actuation highlight the distinct dielectrophoretic responses between cancer cells and PBMCs. Fluorescence images acquired at the outlet region further demonstrate the separation outcome. Under EF-off conditions, (i) MDA-MB-231 cells, (j) MCF-7 cells, and (k) PBMCs predominantly follow the waste outlet route, as shown in the merged image in (l). When the electric field is activated, (m) MDA-MB-231 and (n) MCF-7 cells are redirected toward the CTC outlet streams (only the bottom CTC outlet is shown). In contrast, (o) PBMCs largely remain in the waste outlet stream, although a fraction of the population follows trajectories closer to the sidewalls and enters the CTC outlet streams, contributing to reduced purity in the collected cancer cell fractions. The corresponding merged fluorescence image is shown in (p). All fluorescence images were acquired at the same magnification. Fluorescence signals were recorded in grayscale and are displayed using consistent pseudocolors assigned during image processing (green for MDA-MB231 cells, red for MCF7 cells, and blue for PBMCs) to facilitate visualization and comparison of the different cell populations. Flow direction is from left to right, and the scale bar in panel (a) applies to all panels. While figure illustrates representative snapshots from the solo experiments, the dynamic behavior observed during co-culture and PBMC spike-in experiments is presented in SI Videos S1–S8.

Cancer cells experienced positive dielectrophoretic forces near the tips of the insulating ratchet structures when an AC electric field (210 V_RMS_, 8 kHz) was applied. As a result, the manipulated cells were laterally displaced toward streamlines closer to the channel sidewalls while traversing the DEP region (Fig. 3e and f). In contrast, PBMCs exhibited negligible lateral displacement and largely maintained their original streamlines (Fig. 3g). This enables effective spatial separation between cancer and blood cell populations. Comparable trajectory modulation was observed for both epithelial-like (MCF-7) and mesenchymal-like (MDA-MB-231) phenotypes, demonstrating robust DEP-driven manipulation across heterogeneous cancer cell types.

The applied electric field and flow conditions provided sufficient lateral DEP migration to redirect cancer cells toward the CTC outlets. The performance of the device is sensitive to the balance between hydrodynamic focusing and DEP-induced lateral migration. The initial lateral position of cells entering the DEP region determines the displacement required to reach the CTC-directed streamlines and also influences the electric-field gradient initially experienced by each cell. Insufficient DEP relative to hydrodynamic transport may result in incomplete lateral displacement and passage toward the waste outlet, whereas excessive DEP-induced migration may cause trapping of cells at the high-field-gradient regions near the constriction tips. Thus, hydrodynamic focusing width, electric-field strength, flow rate, cell size, and dielectric properties collectively define the operating window and sensitivity of the present design.

The effect of the applied voltage on cellular integrity was qualitatively assessed using MDA-MB-231 and MCF-7 cells under EF-off, 210 V_RMS_, and 350 V_RMS_ conditions (Fig. S4). At the selected operating condition of 210 V_RMS_, both cell populations underwent DEP manipulation while retaining their overall morphology and clear intracellular fluorescence, with no apparent deterioration relative to the EF-off condition. In contrast, at approximately 350 V_RMS_, fluorescence loss and compromised cellular integrity were observed, including visible extracellular mCherry fluorescence in the surrounding buffer for MCF-7 cells. These observations indicate that the pronounced cellular deterioration observed at the higher voltage was not apparent at the selected 210 V_RMS_ operating condition. However, as GFP and mCherry fluorescence are not direct viability markers, these observations were interpreted as a qualitative assessment of cellular viability.

At the outlet region, fluorescence imaging further confirmed the separation behavior observed in the DEP region. In the absence of an applied electric field, MDA-MB-231 cells, MCF-7 cells, and PBMCs predominantly followed the hydrodynamically focused central streamline and exited through the waste outlet (Fig. 3i–l). This observation is consistent with the dominance of hydrodynamic drag forces under EF-off conditions, where no lateral trajectory modulation occurs.

Upon application of the AC electric field, cancer cells underwent lateral displacement due to positive dielectrophoretic forces generated near the insulating ratchet structures. Consequently, both MDA-MB-231 and MCF-7 cells were redirected toward streamlines near the channel sidewalls and subsequently collected at the designated CTC outlet (Fig. 3m and n). In contrast, the majority of PBMCs remained within the central streamline and continued toward the waste outlet, although a fraction of the population exhibited lateral displacement and entered the CTC outlet streams (Fig. 3o). This partial co-enrichment of PBMCs is attributed to overlapping dielectric and biophysical characteristics between certain PBMC subpopulations and the target cancer cells, contributing to reduced purity in the collected fractions. The merged fluorescence image in Fig. 3p further demonstrates the selective isolation of heterogeneous cancer cell populations from PBMCs. Representative real-time recordings of the co-culture and spike-in PBMC experiments under both EF-on and EF-off conditions are provided in SI Videos S1–S4 and S5–S8.

### Quantitative analysis of cancer cell manipulation and purity

To quantify cell distributions and sample purity, outlet regions were considered. Recorded image frames were imported into ImageJ, where a semi-automated fluorescent cell counting workflow was applied to quantify particles within identical regions of interest (ROIs) positioned downstream of the bifurcation. This workflow consisted of standardized thresholding, ROI selection, and size-based particle detection steps applied identically across all frames, as summarized in Fig. 4 and the SI. Particle counts obtained for each frame and outlet were averaged to determine the mean cell occupancy per frame.

**Fig. 4:**
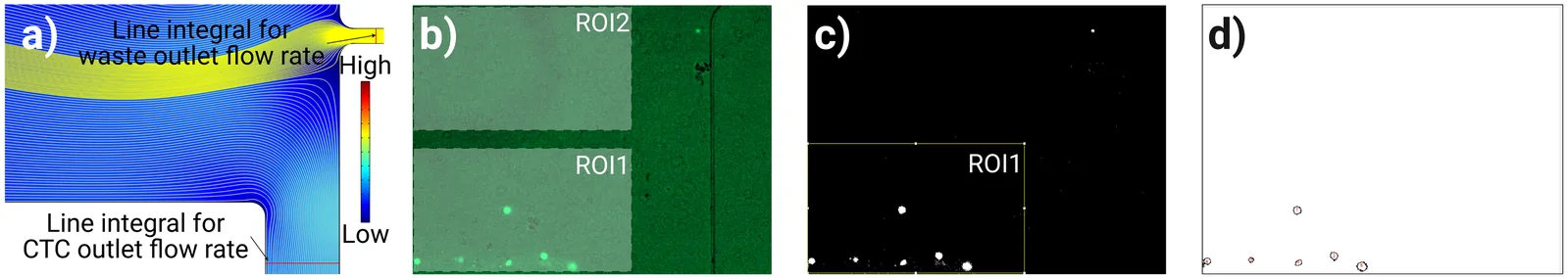
Cell counting methodology. (a) COMSOL Multiphysics 6.2 (COMSOL AB, Sweden) flow simulations showing the separation of sample and waste streams at the outlet region. Waste fluid is directed toward the waste outlet (yellow streamlines), whereas sample fluid exits through the upper and bottom CTC outlets (gray streamlines). Outlet flow rates were obtained by integrating the velocity field along vertical cut lines (line integrals) at each outlet. These values were subsequently used to calculate cell fluxes based on the average cell occupancies per frame (*n*_1,avg_ and *n*_2,avg_) measured within ROI1 and ROI2 in b, separately. Full outlet-region streamline distribution is shown in the SI Fig. S3b. (b) Representative fluorescence image of the same outlet region used for experimental quantification of total MDA-MB-231 cell occupancies (*N*_1_ and *N*_2_) within ROI1 and ROI2 over 50 consecutive frames. (c) Binary threshold image generated in ImageJ showing the defined ROIs used for automated cell detection. Object selection criteria included minimum diameter and circularity thresholds. (d) Final cell detection result, where detected cells are indicated by circular outlines.

Because instantaneous ROI occupancies depend on local residence time, raw occupancies could not be interpreted directly as particle fractions due to unequal outlet flow conditions. In the present microfluidic system, varying sheath and sample flow rates resulted in non-uniform outlet flow distributions, thereby affecting cell residence times within the monitored ROIs. Hence, occupancies were converted into outlet-specific cell fluxes. Local velocities and volumetric flow rates at the ROIs were obtained from laminar-flow COMSOL simulations using the experimental geometry and boundary conditions (Fig. 4a). Outlet flow rates were determined by integrating the velocity field along vertical cross-sections (line integrals) at each outlet. Detailed outlet flow-rate calculations and corresponding COMSOL expressions are provided in Table S1 in the SI. Cell fluxes were subsequently calculated by combining the average number of detected cells per frame within each ROI with the corresponding outlet flow rate.3
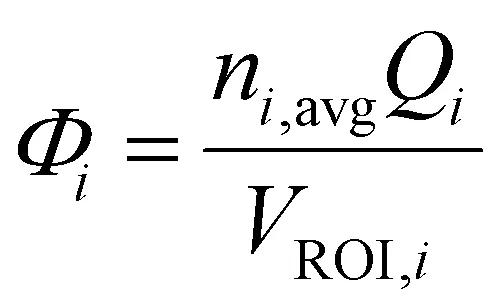
where *Φ*_*i*_ is the cell flux at outlet *i* (cells per min), *n*_*i*,avg_ is the average number of detected cells per frame within ROI_*i*_, *Q*_*i*_ is the corresponding outlet volumetric flow rate (μl min^−1^), and *V*_ROI,*i*_ is the effective observation volume (μl) of the ROI. The geometric volume of each counting ROI was calculated individually as:

4 *V*_ROI,*i*_ = *L*_ROI,*i*_*W*_ROI,*i*_*h*,

where *L*_ROI,*i*_ and *W*_ROI,*i*_ are the measured planar ROI dimensions and *h* = 120 μm is the nominal channel height. Volumes were converted from μm^3^ to μL using 1 μm^3^ = 10^−9^ μL. Because ROI dimensions varied between recordings and outlet regions, *V*_ROI,*i*_ was determined separately for each ROI.

Cancer cell manipulation rate (CMR) was calculated by combining the cancer cell fluxes from the two CTC outlet streams and normalizing them to the total cancer cell flux collected from all outlets:5
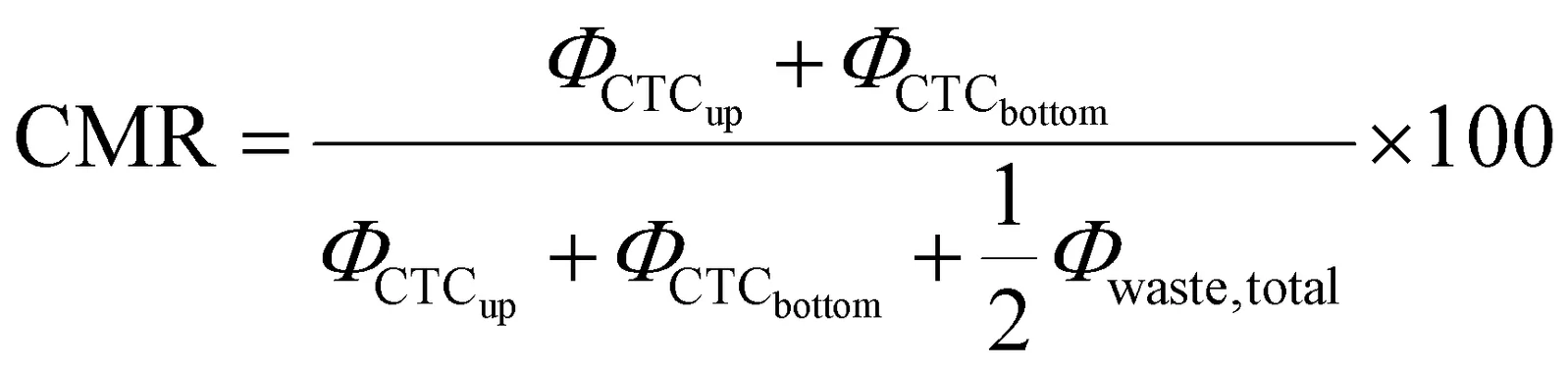


Since the waste outlet was visible in both outlet-region recordings, cells entering it were counted twice during image analysis. To avoid overestimating the waste fraction, the total measured waste flux was divided by two before calculating CMR. CMR was calculated as the combined cancer-cell flux collected from the two CTC outlets relative to the corrected total cancer-cell flux across all outlet streams.

For spike-in PBMC experiments, purity was calculated as the fraction of cancer-cell flux within the combined CTC outlet streams relative to the total collected cell flux in these outlets:6
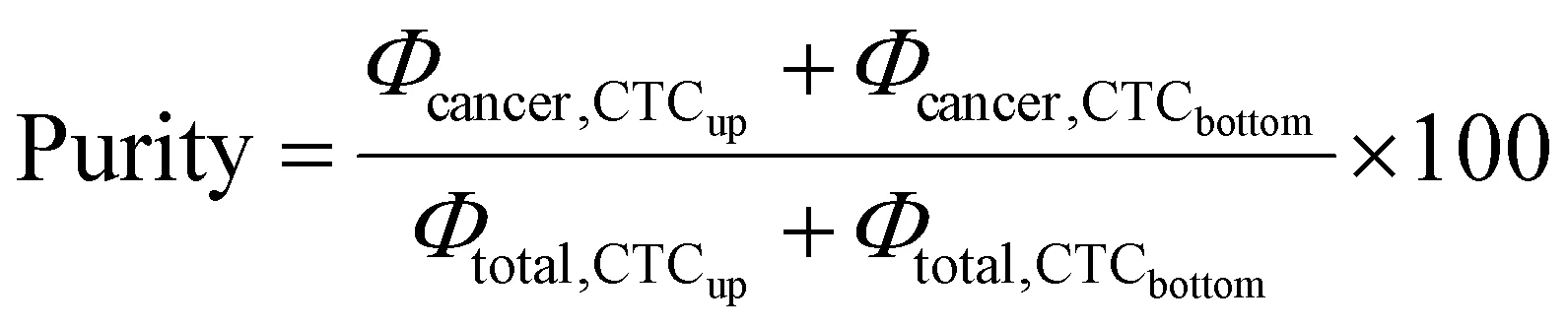


For each independent experiment, cell counts were averaged across the analyzed frames for each ROI and converted to outlet-specific cell fluxes using eqn (3). A single CMR value was then calculated from the resulting average outlet fluxes using eqn (5). Reported CMR values represent the mean ± SD across three independent experiments (*N*_exp_ = 3). A representative calculation showing the mean cell counts, ROI volumes, outlet-specific flow rates, calculated cell fluxes, and experiment-level CMR values is provided in Table S2 of the SI. Image analysis was performed in ImageJ, where binary thresholding was applied to generate segmentation masks for automated cell detection (Fig. 4c). Regions of interest corresponding to each outlet stream were defined, and object selection was performed according to the applied analysis protocol (described in the SI), using size (minimum and maximum area) and circularity criteria to exclude debris and noise. The final detection output consisted of identified cells within each ROI separately (Fig. 4d), enabling consistent and reproducible quantification of outlet-specific cell distributions.

Device performance as sample complexity increases is shown in Fig. 5. First, single-cell-type suspensions of MDA-MB-231 and MCF-7 were used to evaluate the performance under simplified conditions. High and consistent CMR were obtained for both cell lines, with values of (98.3 ± 0.8)% and (99.5 ± 0.5)%. Then, to assess the impact of inter-cell-type interactions, experiments were subsequently performed using a 1 : 1 co-culture of the two cell lines. Under these conditions, the CMR remained comparably high ((98.8 ± 0.3)%), indicating that the presence of a second cancer cell population did not adversely affect manipulation efficiency. Further evaluation was performed by spiking cancer cells into PBMC suspensions at a 1 : 1 cancer cell-to-PBMC ratio, thereby introducing a heterogeneous cellular background and enabling assessment of the platform under increased sample complexity. The CMR in the samples with cancer cells spiked into PBMCs ((98.7 ± 0.4)%) were consistent with both the solo and co-culture experiments. No statistically significant differences were observed in CMRs among solo, co-culture, and spike-in PBMC conditions (Welch's *t*-test, *p* > 0.05), indicating that performance is maintained as sample complexity increases. The comparable CMR obtained for both epithelial-like (MCF-7) and mesenchymal-like (MDA-MB-231) phenotypes also demonstrate the platform's robustness across heterogeneous cancer cell populations.

**Fig. 5:**
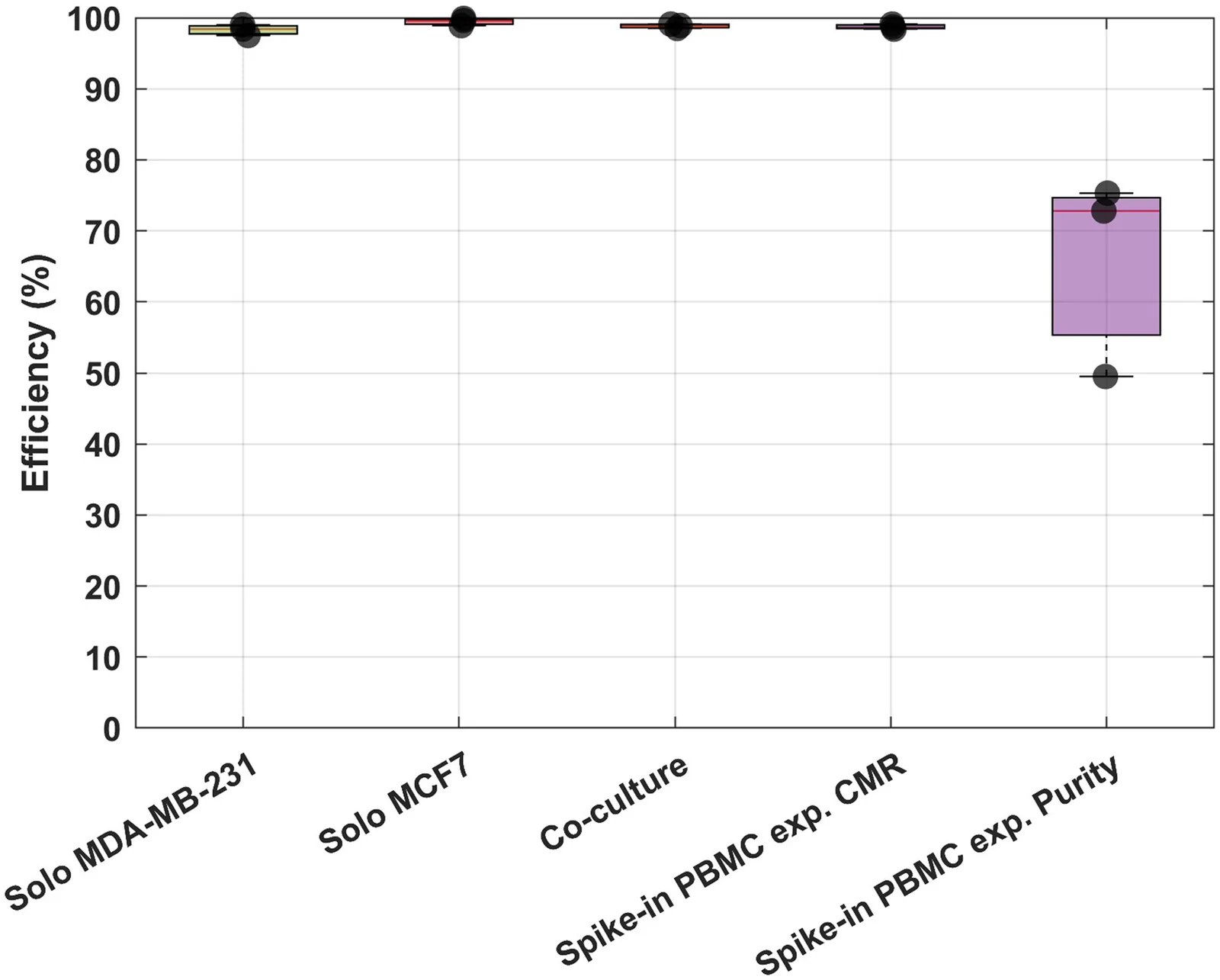
Cancer cell manipulation performance across increasing sample complexity and corresponding purity in spike-in PBMC conditions. Box plots summarizing the CMR of MDA-MB-231 and MCF-7 under three experimental conditions of increasing biological complexity. Solo experiments represent single-cell-type suspensions of each cell line processed independently. Co-culture experiments correspond to a 1 : 1 mixture of MDA-MB-231 and MCF-7 cells, enabling assessment of isolation performance in the presence of inter-cell-type interactions. Spike-in PBMC experiments represent a complex biological matrix in which cancer cells are spiked into PBMCs, mimicking clinically relevant conditions. For the spike-in PBMC case, both CMR (fraction of target cancer cells collected at the designated outlet relative to all outlets) and purity (fraction of recovered cells identified as target cancer cells relative to total collected cells) are reported.

The purity of collected samples was evaluated in spike-in PBMC experiments to assess the separation performance of the device in the presence of heterogeneous blood-cell background. While CMR remained consistently high ((98.7 ± 0.4)%), purity was substantially lower and exhibited greater variability, with a value of ((65.9 ± 14.1)%). The reduced purity was primarily attributed to the co-enrichment of non-target PBMCs which exhibit electrokinetic characteristics that partially overlap with those of the target cancer cells under the applied operating conditions. Donor-to-donor variation in PBMC composition, including differences in the relative abundance of monocytes, may have contributed to the observed variability in purity. Purities of approximately 60–70% have previously enabled cell-resolved analyses such as immunofluorescence and fluorescence *in situ* hybridization (FISH) of CTCs,^68^ while targeted molecular assays can also tolerate residual leukocyte contamination when tumor-specific targets and appropriate background controls are used. The residual PBMC contamination observed here reflects the broader challenge of balancing target-cell enrichment with background-cell depletion in CTC isolation. Further optimization of the constriction and outlet geometries, together with the electrode configuration, could improve the balance between hydrodynamic transport and DEP-induced lateral migration, potentially reducing PBMC co-enrichment while maintaining efficient cancer-cell manipulation for downstream applications that are particularly sensitive to leukocyte contamination.^69^

### Performance comparison with representative CTC enrichment technologies

The performance of the present platform was further compared to representative established and microfluidic CTC enrichment technologies (Table 1). EpCAM-dependent CellSearch system showed markedly different recovery for MCF-7 (89 ± 13%,^70^ 70 ± 10% ^71^) and MDA-MB-231 cells (12 ± 15% ^70^). This difference is consistent with their contrasting EpCAM expression, as epithelial-like MCF-7 cells express high levels of EpCAM, whereas mesenchymal-like MDA-MB-231 cells exhibit low EpCAM expression and are therefore less efficiently captured by EpCAM-dependent enrichment. Similarly, an anti-EpCAM-functionalized polymeric CTC-Chip showed efficient capture of MCF-7 cells (∼90% in PBS), whereas capture of the EpCAM-low MDA-MB-231 cells was only ∼10%, further illustrating the phenotype dependence of EpCAM-based positive selection.^72^ The CTC-iChip, which integrates deterministic lateral displacement, inertial focusing, and magnetophoresis, reported recoveries ranging from 77.8 ± 7.8% for MDA-MB-231 to 98.6 ± 4.3% for SKBR3 cells in its antigen-dependent configuration.^73^ This platform can operate in either positive- or negative-selection modes, both having labeling methodology. ClearCell FX achieved 64.5 ± 12.3% recovery of MCF-7 cells from spiked blood,^74^ while the size- and deformability-based Parsortix PR1 showed size dependent capture, with linear-regression capture slopes of 43.67% for MDA-MB-231, 63.79% for SKBR3 cells,^75^ and 77% for MCF7 cells.^76^ Among DEP-based approaches, ApoStream provides a particularly relevant comparison, as it enables continuous and label-free enrichment based on differences in cellular dielectric properties. ApoStream achieved recoveries of 71.2 ± 1.6% for MDA-MB-231 and 75.4 ± 3.1% for SKOV3 cells in a PBMC background.^77^

**Table 1:** Comparison of the present iDEP platform with representative established and microfluidic CTC enrichment technologies^a^

Platform	Principle	Labeling	Sample/cell model	Performance	Ref.
CellSearch	Immunomagnetic enrichment	EpCAM-dependent	MDA-MB-231 & MCF-7 spiked into healthy-donor blood	MDA-MB-231: 12 ± 15% recovery; MCF-7: 89 ± 13% & 70 ± 10% recovery	70 and 71
CTC-Chip	Affinity capture on microposts	EpCAM-dependent	MDA-MB-231 & MCF-7 in PBS solution	MDA-MB-231: ∼10% recovery; MCF-7: ∼90% recovery	72
CTC-iChip	DLD, inertial focusing, and magnetophoresis	EpCAM/CD45 dependent	Cancer cell lines spiked into whole blood	SKBR3: 98.6 ± 4.3%; PC3–9: 89.7 ± 4.5%; MDA-MB-231: 77.8 ± 7.8% recovery	73
ClearCell FX	Inertial microfluidics	Label-free	MCF-7 spiked into whole blood	MCF-7: 64.5 ± 12.3% recovery	74
Parsortix PR1	Size- and deformability-based	Label-free	MDA-MB-231 & MCF-7 spiked into healthy-donor blood	Capture slopes: MDA-MB-231, 43.67%; MCF-7, ∼77%	75 and 76
ApoStream	DEP field-flow fractionation	Label-free	MDA-MB-231 & SKOV3 spiked into PBMCs from healthy-donor blood	MDA-MB-231: 71.2 ± 1.6% recovery; SKOV3: 75.4 ± 3.1% recovery	77
Present platform	Insulator-based DEP and hydrodynamic focusing	Label-free	MCF-7 & MDA-MB-231 individually and as heterogeneous mixtures, including PBMC background	CMR >98% for both cancer-cell populations; purity 65.9 ± 14.1% in PBMC-spiked experiments^a^	—

a The definitions of recovery and other performance metrics, cell models, spike levels, sample matrices, enrichment strategies, and experimental protocols vary among these studies. For the present iDEP platform, the cancer-cell manipulation rate (CMR) is an outlet-based metric calculated from the relative cancer-cell fluxes detected at the CTC and waste outlets. DLD: deterministic lateral displacement.

In comparison, the present iDEP platform achieved a cancer-cell manipulation rate (CMR) above 98% for both epithelial-like MCF-7 and mesenchymal-like MDA-MB-231 cells under a common operating condition, including experiments performed in a PBMC background. Since CMR is an outlet-based metric and does not represent absolute inlet-to-output recovery, the present results demonstrate the ability of the iDEP approach to manipulate phenotypically contrasting breast cancer-cell populations without relying on tumor-specific surface markers. Importantly, DEP exploits differences in cellular dielectric properties and can therefore discriminate between cells of similar size that exhibit different electrical responses, a distinction that cannot be achieved by size-based separation alone. This provides an additional separation dimension that may be advantageous when target and background cell populations have overlapping size distributions. Additionally, the current platform operates at a sample flow rate of 5 μL min^−1^, which is sufficient for proof-of-concept characterization but remains below the throughput required for processing clinically relevant blood volumes. Increasing throughput will be necessary for clinical translation.^31^ This could potentially be achieved by parallelization of multiple separation channels or multiplexed device architectures while maintaining the hydrodynamic focusing and electric-field conditions required for selective cell manipulation.

## Conclusions

In this study, a continuous-flow iDEP microfluidic platform for label-free enrichment of phenotypically heterogeneous breast cancer cell populations is demonstrated. By integrating hydrodynamic focusing with localized electric-field gradients generated by insulating microstructures, MCF-7 epithelial-like and MDA-MB-231 mesenchymal-like breast cancer cells were successfully directed toward the designated cancer-cell outlets under a common operating condition of 8 kHz and 210 V_RMS_. This behavior was maintained when the two cancer-cell populations were processed as a heterogeneous mixture and subsequently in the presence of PBMCs. Across these experimental conditions, more than 98% of the detected cancer-cell outlet flux was directed toward the designated cancer-cell outlets, while a cancer-cell purity of approximately 66% was achieved in the PBMC-containing experiments.

The enrichment of epithelial-like and mesenchymal-like breast cancer cells under the iDEP operating condition combined with the hydrodynamic focusing addresses an important challenge associated with CTC phenotypic heterogeneity and the limitations of phenotype-specific affinity markers. The PBMC spike-in experiments used here represent an intermediate model between cancer-cell suspensions and clinically representative blood samples. Patient-derived samples introduce additional complexity, including lower target-cell abundance, and greater variability in CTC size, phenotype, and dielectric properties.

Overall, these findings establish a proof of concept for label-free enrichment of heterogeneous cancer-cell populations and support the potential of iDEP as a complementary approach for microfluidic liquid-biopsy sample preparation. Further development should focus on increasing throughput and purity, evaluating scalability and parallelization, and validating the platform with rare cancer cells in patient-derived blood samples. Integration with downstream molecular analyses, including immunophenotyping, sequencing, and single-cell characterization, will be important for establishing its applicability in clinically relevant liquid-biopsy workflows.

## Author contributions

Conceptualization, G. Ö., and P. E. B.; investigation, G. Ö., I. N. U., E. Y., and J. K.; methodology, G. Ö., I. N. U., J. K., D. B., G. R. P., and P. E. B.; validation, G. Ö., I. N. U., and E. Y.; formal analysis, G. Ö. and G. R. P.; writing – original draft preparation, G. Ö.; writing – review and editing, G. Ö., J. K., J. W. M. M., P. tD., G. R. P., and P. E. B.; visualization, G. Ö., I. N. U, and E. Y.; resources, D. B., K. D., J. W. M. M., P. tD., and P. E. B.; supervision, G. Ö., K. D., P. tD., G. R. P., and P. E. B.; funding acquisition, J. W. M. M., P. tD., and P. E. B. All authors have read and agreed to the published version of the manuscript.

## Conflicts of interest

There are no conflicts to declare.

## Abbreviations

AC: Alternating current
CMR: Cancer-cell manipulation rate
CTC: Circulating tumor cell
DEP: Dielectrophoresis
DLD: Deterministic lateral displacement
eDEP: Electrode-based dielectrophoresis
EF: Electric field
EMT: Epithelial–mesenchymal transition
GFP: Green fluorescent protein
iDEP: Insulator-based dielectrophoresis
PBMC: Peripheral blood mononuclear cell
PBS: Phosphate-buffered saline
PDMS: Polydimethylsiloxane
ROI: Region of interest
RMS: Root mean square

## Supplementary Material

AN-OLF-D6AN00976J-s001

AN-OLF-D6AN00976J-s002

AN-OLF-D6AN00976J-s003

AN-OLF-D6AN00976J-s004

AN-OLF-D6AN00976J-s005

AN-OLF-D6AN00976J-s006

AN-OLF-D6AN00976J-s007

AN-OLF-D6AN00976J-s008

AN-OLF-D6AN00976J-s009

## Data Availability

Supplementary information (SI) is available. See DOI: https://doi.org/10.1039/d6an00976j. The datasets generated and analyzed in this study are available at the 4TU.ResearchData repository (https://data.4tu.nl/private_datasets/7xiArqx4sVk-F2RyCkWgBUxpFZg972YUaAQ5RRr1CrM).
