# Supplementary material for: Label-free isolation of heterogeneous breast cancer cell populations via insulator-based dielectrophoresis
Source: Analyst. 2026 Nov 9. Online ahead of print. doi: 10.1039/d6an00976j (PMC13761689; doi:10.1039/d6an00976j)
Supplement: Supplementary file 1 [file AN-OLF-D6AN00976J-s001.pdf]

Supplementary Information (SI) for Analyst.
This journal is © The Royal Society of Chemistry

Supplementary Information

Label-free Isolation of Heterogeneous Cancer Cell Populations via Insulator-Based Dielectrophoresis

Gürhan Özkayar,[a] Ismene N. Usman,[a] Ece Yakın,[a] Jaco Kraan,[b] Kristen David,[a] Duco Bosma,[a] John W.M. Martens,[b] Peter ten Dijke,[c] Georg R. Pesch,[d] and Pouyan E. Boukany*[a]

[a] *Department of Chemical Engineering, Delft University of Technology, Delft, the Netherlands, E-mail: p.e.boukany@tudelft.nl*
[b] *Department of Medical Oncology, Erasmus MC Cancer Institute, Erasmus University Medical Center, Rotterdam, the Netherlands*
[c] *Oncode Institute and Department of Cell Biology, Leiden University Medical Center, Leiden, the Netherlands*
[d] *Institute of Chemical, Environmental and Bioscience Engineering, TU Wien, Vienna, Austria*

Supplementary Videos

The supplementary videos provide representative real-time fluorescence microscopy recordings of the co-culture and spike-in PBMC experiments under both EF-on and EF-off conditions. Videos were acquired at the DEP and outlet regions to illustrate the dynamic trajectory modulation and separation of heterogeneous cancer cell populations.

- **Video S1.** MDA-MB-231-LifeAct GFP and MCF-7-mCherry co-culture experiment recorded at the DEP region under EF-on conditions, demonstrating DEP-induced lateral migration of both breast cancer cell populations in the separation region.
- **Video S2.** MDA-MB-231-LifeAct GFP and MCF-7-mCherry co-culture experiment recorded at the DEP region under EF-off conditions, showing cell trajectories in the absence of DEP-induced lateral migration.
- **Video S3.** MDA-MB-231-LifeAct GFP and MCF-7-mCherry co-culture experiment recorded at the outlet region under EF-on conditions, demonstrating the routing of both breast cancer cell populations toward the designated CTC outlets following DEP manipulation.
- **Video S4.** MDA-MB-231-LifeAct GFP and MCF-7-mCherry co-culture experiment recorded at the outlet region under EF-off conditions, showing the outlet distribution of the cancer cells in the absence of DEP manipulation.
- **Video S5.** Spike-in PBMC experiment containing MDA-MB-231-LifeAct GFP, MCF-7-mCherry, and PBMCs, recorded at the DEP region under EF-on conditions. The recording demonstrates DEP-induced lateral migration of the breast cancer cells while the majority of PBMCs exhibit substantially weaker lateral displacement.
- **Video S6.** Spike-in PBMC experiment containing MDA-MB-231-LifeAct GFP, MCF-7-mCherry, and PBMCs, recorded at the DEP region under EF-off conditions, showing the trajectories of the heterogeneous cell population in the absence of DEP manipulation.
- **Video S7.** Spike-in PBMC experiment containing MDA-MB-231-LifeAct GFP, MCF-7-mCherry, and PBMCs, recorded at the outlet region under EF-on conditions, demonstrating preferential routing of the breast cancer cells toward the CTC outlets while the majority of PBMCs remain directed toward the central waste outlet.
- **Video S8.** Spike-in PBMC experiment containing MDA-MB-231-LifeAct GFP, MCF-7-mCherry, and PBMCs, recorded at the outlet region under EF-off conditions, showing the outlet distribution of the heterogeneous cell population in the absence of DEP manipulation.

S1. Numerical simulation and model validation

Numerical simulations were performed to verify the hydrodynamic and electrical operating conditions of the final microfluidic device and to support the interpretation of the experimental results.

Finite-element simulations were performed using COMSOL Multiphysics 6.2 (COMSOL AB, Sweden) to evaluate fluid flow and electric field distributions within the final microfluidic device geometry. The simulations employed two coupled physics interfaces: Creeping Flow and Electric Currents (frequency domain). Material properties and boundary conditions were selected to reproduce the experimental operating conditions.

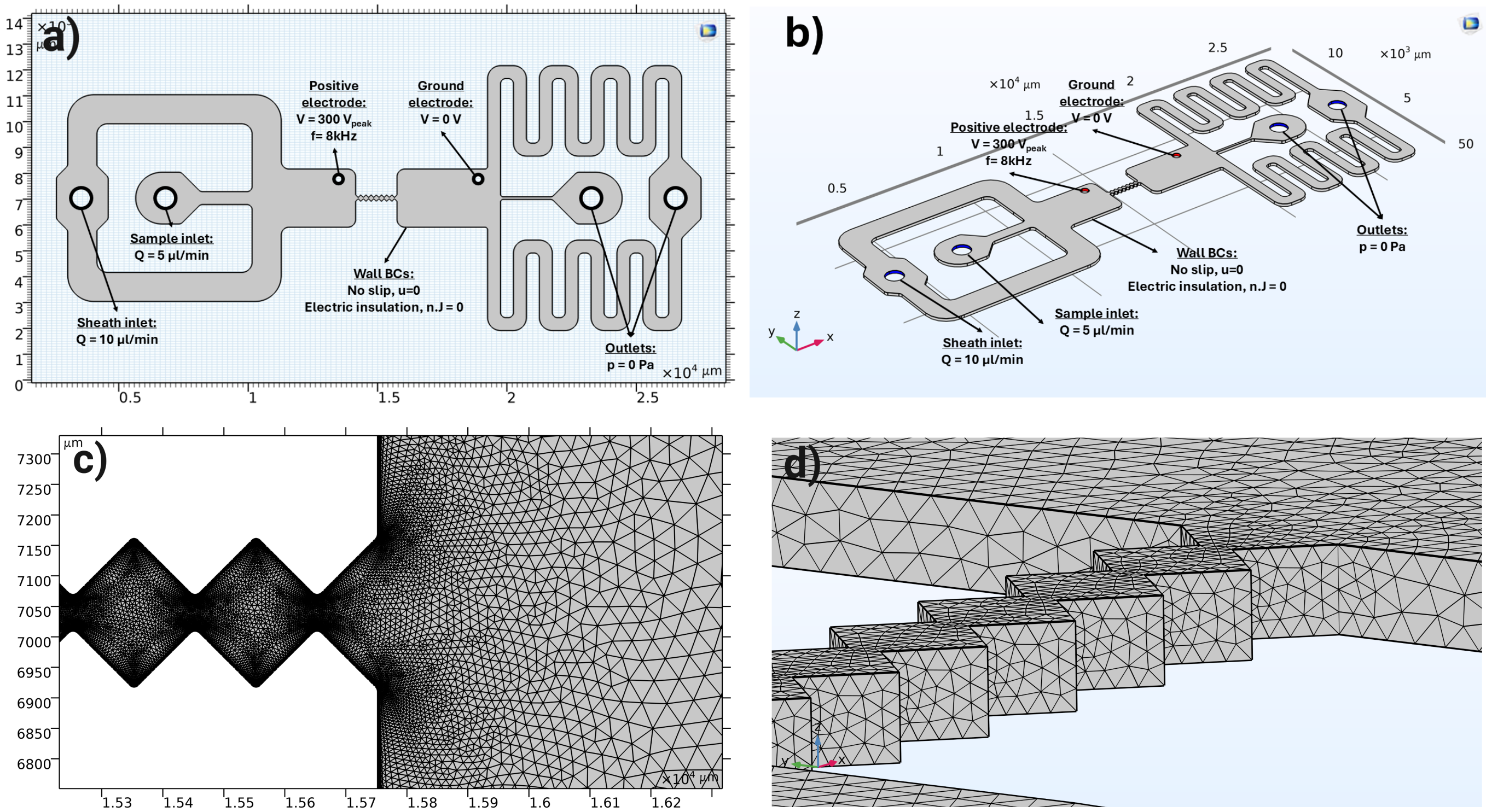

Fig. S1 Computational models used for finite-element simulations. (a) Two-dimensional geometry of the microfluidic device used for fluid flow and electric field simulations. The sample and sheath inlets were prescribed at flow rates of 5 and 10 μL min^{-1}, respectively, implemented in the planar 2D model as the corresponding equivalent average inlet velocities. The outlet boundaries were set to $p = 0$ Pa, and the channel walls were treated as no-slip boundaries. For the electrical model, an AC voltage was applied to the inlet electrode, while the outlet electrode was held at ground ($V = 0$); the remaining channel boundaries were treated as electrically insulating. (b) Three-dimensional geometry used to evaluate the electric field distribution across the 120 μm channel height within the DEP region. (c) User-controlled computational mesh of the two-dimensional model generated using free triangular elements with the predefined Extra Fine Fluid Dynamics mesh sequence. Three boundary-layer elements with a thickness factor of 2.6 were applied along all channel walls to improve the resolution of near-wall flow gradients. Local mesh refinement around the constriction region and insulating obstacle tips was used to resolve the steep electric field gradients responsible for dielectrophoretic manipulation.

Fluid flow was modeled using the Creeping Flow interface under steady-state, incompressible conditions. The sample and sheath inlet boundary conditions were prescribed as volumetric flow rates of 5 and 10 μL min^{-1}, respectively. Because the computational model was two-dimensional, the prescribed volumetric flow rates were converted to equivalent average inlet velocities by assuming the inlet to represent the lateral surface of a cylindrical channel. Accordingly, the effective inlet area was approximated as $A = 2\pi rh$, where $r = 400$ μm is the inlet radius and $h = 120$ μm is the channel height. A pressure boundary condition ($P = 0$ Pa) was applied at the outlet, while no-slip boundary conditions were imposed on all channel walls. The applicability of the Creeping Flow interface was verified by estimating the characteristic Reynolds number of the flow. The Reynolds number was calculated as:

$$\mathrm{Re} = \frac{\rho U D_h}{\mu}, \tag{1}$$

where ρ is the fluid density, U is the average inlet velocity, D_h is the hydraulic diameter of the inlet channel, and μ is the dynamic viscosity of the DEP buffer. The average inlet velocity was determined from the total volumetric flow rate according to:

$$U = \frac{Q}{A}, \tag{2}$$

where Q is the total inlet flow rate and A is the effective inlet area used to prescribe the inlet boundary condition. The hydraulic diameter of the rectangular main channel was calculated as:

$$D_h = \frac{2wh}{w+h}, \tag{3}$$

where $w = 2250$ μm is the main channel width and $h = 120$ μm is the channel height. Using a total flow rate of 15 μL min^{-1}, DEP buffer fluid properties ($\rho = 1000$ kg m^{-3} and $\mu = 1.0 \times 10^{-3}$ Pa s), and the calculated average inlet velocity, the Reynolds number was estimated to be approximately 0.21. This confirms that viscous forces dominate over inertial effects and validates the use of the Creeping Flow interface for the present simulations.

The electric field distribution was calculated using the Electric Currents interface in the frequency domain. One electrode was assigned a ground potential ($V = 0$), whereas the opposing electrode was excited with an AC voltage ranging from 0 to 500 V_{peak} (350 V_{RMS}) at 8 kHz. In COMSOL, the frequency-domain solver uses the peak voltage amplitude as the excitation input. Electrically insulating boundary conditions were applied to all PDMS and channel-wall boundaries. The electrical conductivity of the fluid domain was set to 2 mS m^{-1} to match the DEP buffer used experimentally. The resulting electric field distributions were subsequently used to evaluate the electric field strength and the electric field gradient, within the DEP region.

Table S1 Outlet flow-rate calculations obtained from COMSOL line integrations of outlet velocity profiles under applied sample and sheath inlet flow rates of 5 and 10 μL min^{-1}, respectively. A channel height of $h = 120$ μm was used, corresponding to the fabricated microchannel height.

**Outlet**	**Outlet width (Cut-line length)**	**Line integration formula**	**COMSOL expression**	**Flow rate (μl/min)**
CTC outlet-up	700 μm	$Q_i = h \int_{\Gamma_i} u_n \, d\Gamma$	spf.U*channelheight	7.04
Waste outlet	150 μm	$Q_i = h \int_{\Gamma_i} u_n \, d\Gamma$	spf.U*channelheight	0.82
CTC outlet-bottom	700 μm	$Q_i = h \int_{\Gamma_i} u_n \, d\Gamma$	spf.U*channelheight	7.04

The imposed total inlet flow rate in the simulations was 15.00 μL min^{-1}, corresponding to the combined sample and sheath inlet flow rates. COMSOL line integrations yielded a total outlet flow rate of 14.90 μL min^{-1} with a relative error of approximately 0.67%. The close agreement between the imposed inlet and calculated outlet flow rates confirms mass conservation throughout the computational domain and validates the COMSOL simulations used to compute outlet-specific flow rates and cell-fluxes.

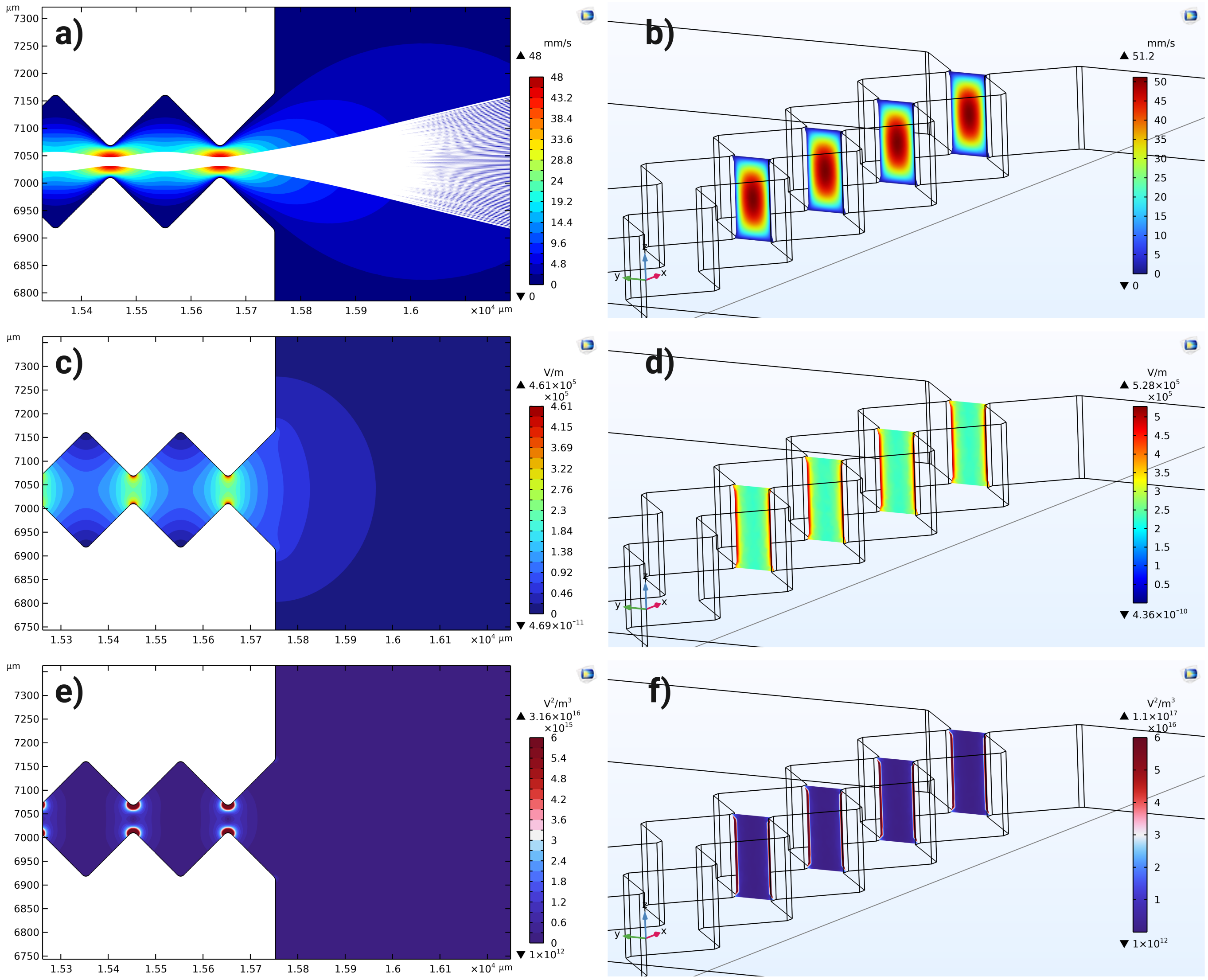

Fig. S2 Finite-element simulation results for the final microfluidic device under the experimental operating conditions. (a) Two-dimensional flow velocity distribution demonstrating stable hydrodynamic focusing generated by sample and sheath flow rates of 5 and 10 μL min^{-1}, respectively. (b) Three-dimensional vertical cross-sectional view of the flow velocity profile showing the velocity distribution throughout the 120 μm channel height. (c) Two-dimensional electric field strength distribution within the DEP region at an applied voltage of 300 V_{peak} (210 V_{RMS}) and 8 kHz. (d) Three-dimensional vertical cross-sectional view of the electric field strength demonstrating a nearly uniform electric field distribution throughout the channel height, except in the immediate vicinity of the insulating obstacle surfaces. (e) Two-dimensional distribution of the electric field gradient, $\nabla(E^2)$, relevant to dielectrophoretic force generation. (f) Three-dimensional vertical cross-sectional view of $\nabla(E^2)$ showing that the simulated electric field gradients locally exceed approximately 10^{12} V^2 m^{-3}. The resulting cell displacement depends on the balance between DEP-induced migration and hydrodynamic transport. Plotted voltage quantities are based on peak values.

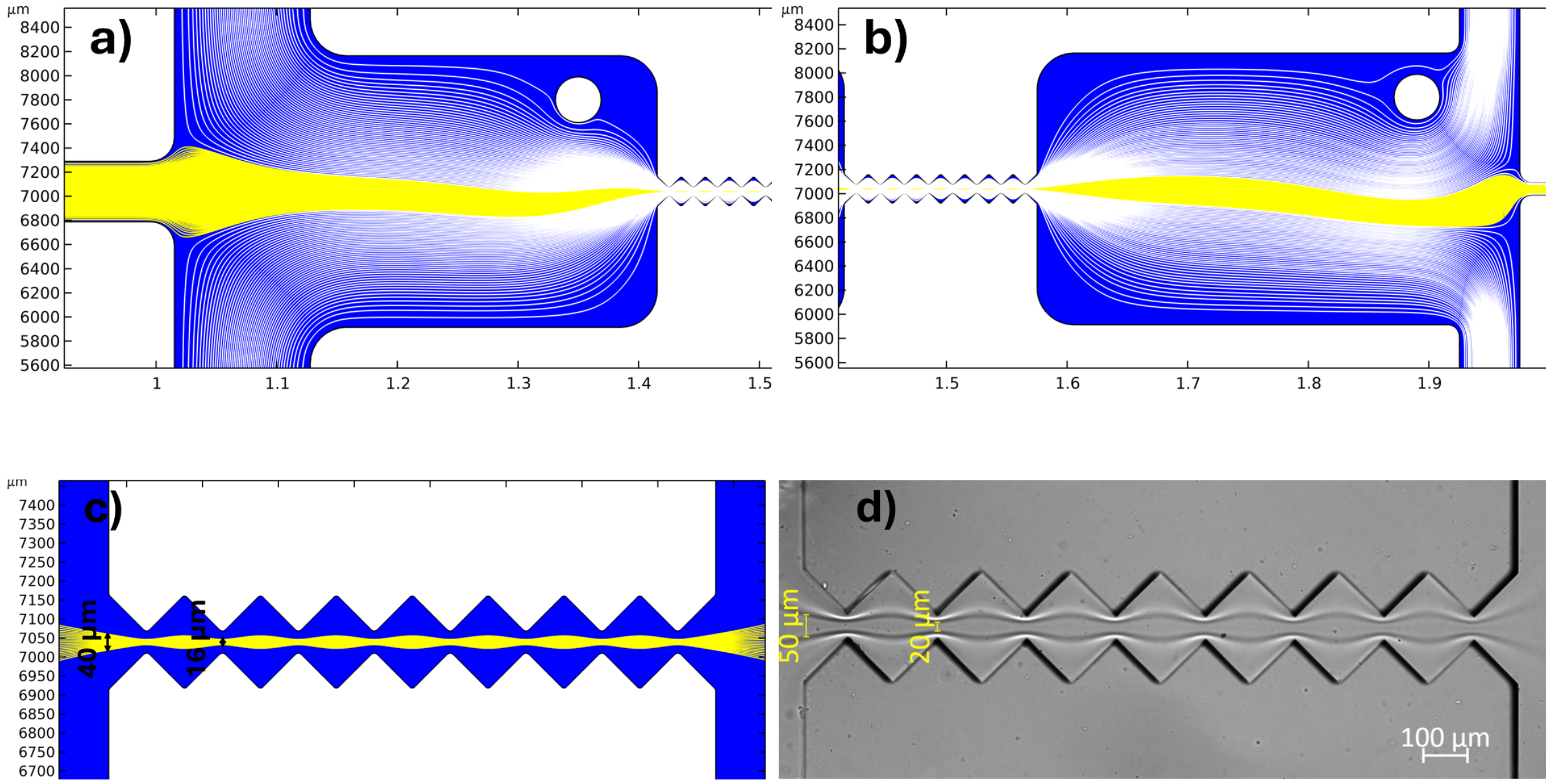

Fig. S3 Numerical and experimental characterization of hydrodynamic focusing through the microfluidic device. (a) Simulated flow streamlines from the sample and sheath-flow inlets toward the DEP region, illustrating hydrodynamic focusing of the sample stream before entering the DEP constriction. Yellow streamlines represent the cell-carrying inlet flow, while white streamlines represent the sheath flows. (b) Simulated flow streamlines through the DEP region and toward the outlets, showing the evolution of the focused cell-carrying stream downstream of the constriction. (c) Representative numerical simulation of the focused cell-carrying stream in the DEP region, showing a reduction in stream width from approximately 40 μm at the entrance of the DEP region to approximately 16 μm at the narrowest section of the constriction. (d) Representative bright-field experimental image showing corresponding cell-stream widths of approximately 50 μm and 20 μm, respectively. Close agreement between the simulated and experimentally observed stream widths supports the predicted hydrodynamic focusing behavior. The resulting narrow stream entering the DEP region confines the cells to a restricted range of initial lateral positions before DEP-induced migration.

S2. Fluorescent cell counting workflow used for multiple images and image stacks in ImageJ/Fiji

1. Open or import the experiment file (`.czi`), which represents an image stack, into ImageJ using Fiji.

    - In the import options, change the color mode to `Grayscale`. Cells may be stained with different fluorescent dyes (e.g., green, red, blue).

2. Convert the image stack type to 8-bit (`Image` → `Type` → `8-bit`).

3. Adjust the threshold of the image stack (`Image` → `Adjust` → `Threshold`).

    - Ensure the `Dark Background` option is checked. Set the method to `Default` and select the `Red` overlay.
    - Adjust the threshold bar until all fluorescently stained cells are fully included within the threshold range (0–255) while keeping the background as dark as possible. Note that the threshold value may vary between datasets due to differences in fluorescence intensity.
    - Click `Apply`. In the `Convert Stack to Binary` window, select the `Default` method and ensure the `Dark Background` option is enabled.
    - If the option `Calculate threshold for each image` is checked, uncheck it. Click `OK` and close the threshold window.

4. Add the region of interest (ROI).

    - Go to `Analyze` → `Tools` → `ROI Manager`.
    - In the ROI Manager window, select `More` → `Specify`. Define the rectangle width and height, and adjust the x, y coordinates.
    - Check the option `Scaled units (microns)` and leave other options unchecked. Click `OK`.
    - Enable `Show All` in the ROI Manager window.
    - To display the ROI in every image of the stack, select `More` → `Options` and uncheck `Associate "Show All" ROIs with slices`.
    - Ensure that the ROI is selected in the ROI Manager before proceeding.

5. Click `Analyze` → `Measure` to record parameters such as area and mean value. Close the measurement window afterward without saving.

6. Perform particle analysis by selecting `Analyze` → `Analyze Particles`.

    - Set the particle size range (minimum and maximum) based on the expected projected cell area in the acquired images. For the cell lines used in this study, a range of 10–10,000 px^2 was found to provide accurate cell detection while excluding most debris.
    - Set the circularity to 0.10–1.00. The circularity threshold may be adjusted after visual inspection of the segmentation results to ensure that individual cells are accurately identified while excluding debris, fragmented cells, and cell aggregates.
    - Select `Outlines` in the `Show` option.
    - Enable `Summarize` and optionally `Exclude on Edges`. Leave other options unchecked and process all images in the stack.

7. The `Summary` window displays parameters such as image index, particle count, total area, and average size for each frame. The total number of counted cells can be calculated manually or obtained directly from the final frame of the image stack.

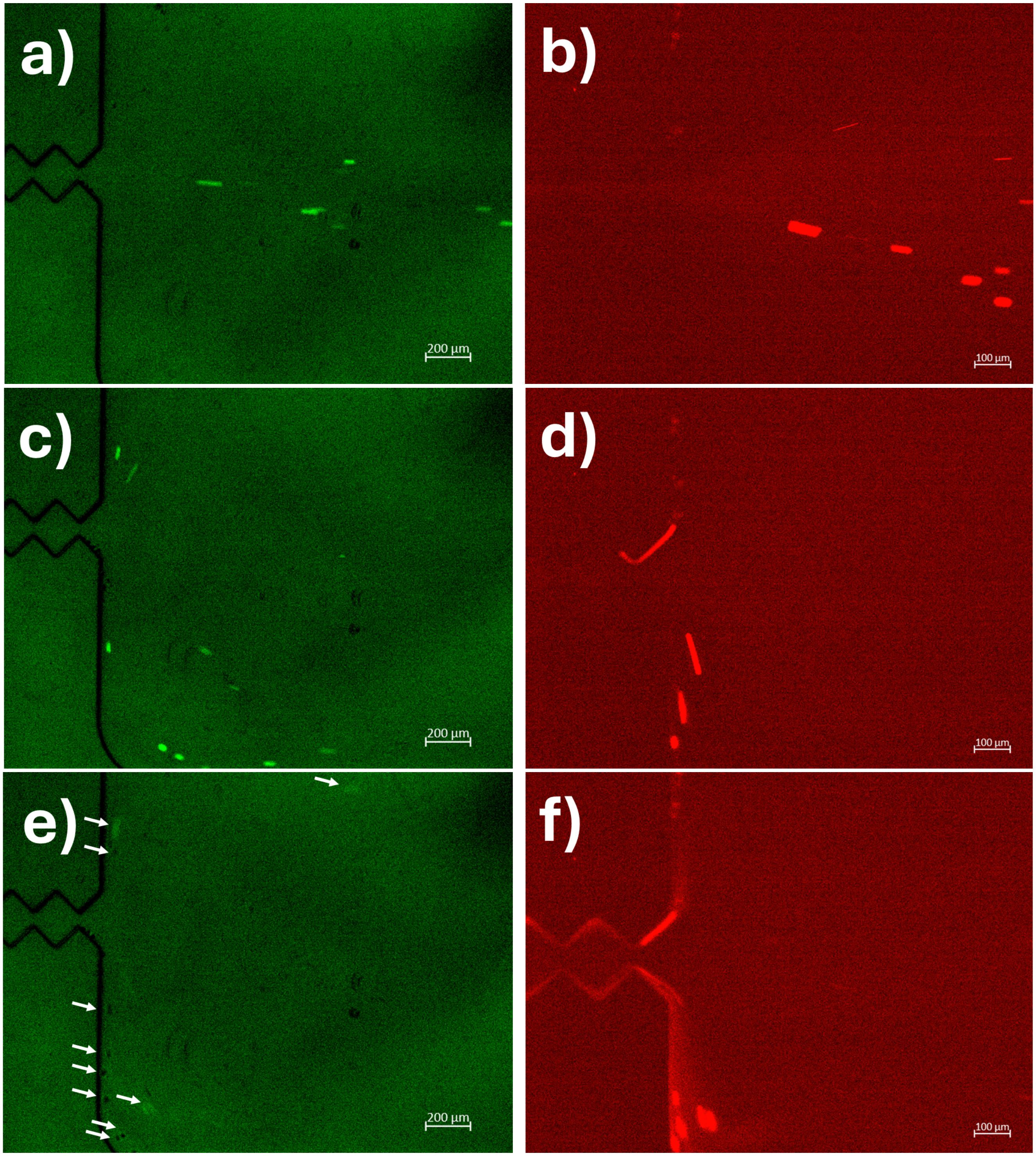

Fig. S4 Qualitative assessment of cellular integrity under different applied voltages. Representative fluorescence images of MDA-MB-231-GFP (a,c,e) and MCF-7-mCherry (b,d,f) cells in the DEP region under EF-off (a,b), 210 V_{RMS} (c,d), and 350 V_{RMS} (e,f) conditions. At 210 V_{RMS}, both cell populations experienced DEP manipulation while retaining clear intracellular fluorescence, with no apparent deterioration compared with the EF-off condition. At approximately 350 V_{RMS}, cancer cells remained responsive to DEP but showed fluorescence loss and compromised cellular integrity. MDA-MB-231 cells exhibiting substantial fluorescence loss are indicated by white arrows in (e), and extracellular mCherry fluorescence in the surrounding buffer is clearly visible for MCF-7 cells in (f). MDA-MB-231 images were acquired using the GFP channel at 5× magnification (scale bars: 200 μm), and MCF-7 images using the mCherry channel at 10× magnification (scale bars: 100 μm).

Table S2 Representative calculation of cell flux and cancer-cell manipulation rate (CMR) for three independent solo MDA-MB-231 experiments. Mean cell counts per frame (n_{avg}), ROI volumes (V_{ROI}), corresponding volumetric flow rates (Q), calculated cell fluxes (Φ), and experiment-level CMR values are shown.

**Condition**	**Exp.**	**Outlet/ROI**	n_{avg}	V_{ROI} (μL) ($L_{ROI} * W_{ROI} * h * 10^{-9}$)	Q (μL min^{-1})	Φ (cells min^{-1})	**CMR (%)**
Solo MDA-MB-231	1	CTC bottom	9.10	0.09438	7.04	678.7879	99.0244%
		CTC up	-	-	7.04	-	
		Waste	0.50	0.061308	0.82	6.687545	
	2	CTC bottom	4.60	0.087264	7.04	371.1038	98.4336%
		CTC up	-	-	7.04	-	
		Waste	0.47	0.0648	0.82	5.90535	
	3	CTC bottom	1.63	0.09288	7.04	123.8013	97.5271%
		CTC up	-	-	7.04	-	
		Waste	0.27	0.06966	0.82	3.139056	
						**Mean ± SD:**	**98.3 ± 0.8**

Table S2 provides the MDA-MB-231 experiments as a representative example of the cell-counting, cell-flux, and CMR calculation procedure. For each independent experiment, the mean cell count per frame (n_{avg}) was calculated for each region of interest (ROI) from a total of 30 frames in the solo and co-culture experiments and 50 frames in the PBMC spike-in experiments. The geometric volume of each ROI (V_{ROI}) was calculated individually as $V_{\mathrm{ROI}} = L_{\mathrm{ROI}} W_{\mathrm{ROI}} h$, where L_{ROI} and W_{ROI} are the planar ROI dimensions and h is the channel height. The ROI dimensions were measured in pixels in ImageJ and converted to μm using the calibrated image scale. The resulting ROI volume, calculated in μm^3, was subsequently converted to μL. The cell flux was calculated as $\Phi = n_{\mathrm{avg}} Q / V_{\mathrm{ROI}}$, where Q is the corresponding COMSOL-derived volumetric flow rate. A single CMR value was subsequently calculated for each independent experiment from the corresponding outlet-specific cell fluxes using Eq. 5. Reported CMR values represent the mean ± SD across three independent experiments ($N_{\mathrm{exp}} = 3$); individual frames were not treated as independent replicates. The same calculation procedure was applied to all experimental conditions, and the underlying raw data for the other experiments are provided in a public repository with a DOI.
